# Midlife cardiovascular health factors as predictors of retirement age, work-loss years, and years spent in retirement among older businessmen

**DOI:** 10.1038/s41598-023-43666-x

**Published:** 2023-10-02

**Authors:** Markus J. Haapanen, Timo Törmäkangas, Monika E. von Bonsdorff, Arto Y. Strandberg, Timo E. Strandberg, Mikaela B. von Bonsdorff

**Affiliations:** 1 Department of General Practice and Primary Health Care, University of Helsinki and Helsinki University Hospital, Helsinki, Finland; 2 Folkhälsan Research Centre, Helsinki, Finland; 3 Department of Medical Epidemiology and Biostatistics, Karolinska Institutet, Stockholm, Sweden; 4 Gerontology Research Center and Faculty of Sport and Health Sciences, University of Jyväskylä, Jyväskylä, Finland; 5 Management and Leadership, Jyväskylä University School of Business and Economics, University of Jyväskylä, Jyväskylä, Finland; 6 Department of Medicine, University of Helsinki and Helsinki University Hospital, Helsinki, Finland; 7 Centre for Life Course Health Research, University of Oulu, Oulu, Finland

**Keywords:** Cardiovascular biology, Risk factors, Epidemiology

## Abstract

Cardiovascular disease (CVD) is one of the leading causes of premature retirement. However, the relationship between CVD risk factors and workforce participation is not well known. We studied the relationship between midlife CVD risk, age at retirement, work-loss years, and survival in retirement. Middle-aged Finnish men (initial n = 3490, mean age = 47.8 years) were assessed for CVD risk factors and general health in the 1970s. They worked as business executives and provided information on their retirement status in the year 2000. Survival was followed up to the 9th decade of life with a follow-up of up to 44 years. Work-loss years were calculated as death or retirement occurring at age ≤ 65 years. Smoking, body mass index, and alcohol use were used as covariates, excluding models of CVD risk, which were adjusted for alcohol use only. Higher risk of 10-year fatal CVD was associated with 0.32 more years (relative risk < 1 vs. 1, covariate-adjusted β = 0.32, 95% CI = 0.13, 0.53) of work-loss. Higher risk of 5-year incident (covariate-adjusted time-constant HR = 1.32, 95% CI = 1.19, 1.47) and 10-year fatal (covariate-adjusted time-dependent HR = 1.55, 95% CI = 1.30, 1.85) CVD in midlife were associated with fewer years spent in retirement. Poorer self-rated health and physical fitness and higher levels of triglycerides were associated with increased hazard of earlier retirement, more work-loss years, and fewer years spent in retirement. Poorer health and greater midlife CVD risk may be associated with earlier exit from the workforce and fewer years spent in retirement. Management of CVD risk in midlife may support people to work longer.

## Introduction

Cardiovascular disease (CVD), a leading cause of premature mortality worldwide^1^, is associated with poorer workforce participation^2^ among older employees by increasing the risk of transitioning into disability pension^3,4^ and exiting the labour force early^5^. It bears high costs to societies, amounting to a quarter of health-related productivity losses in the European Union, with estimates of 54 billion in the year 2019^6^. Whether CVD risk factors, including elevated blood pressure, serum lipids, and blood glucose among others, would also associate with workforce participation among individuals of working age, is poorly understood^7,8^. Understanding this association is becoming increasingly important as more individuals are expected to work longer in the future, regardless of coexisting chronic illnesses.

A recent meta-analysis highlighted the need to consider prior health when studying the relationship between retirement timing and mortality^9^. This can only be examined with follow-up studies started at earlier stages of the life course, with subjectively and objectively measured data on health at working age and in retirement, as well as data on mortality. There are few previous studies that have investigated the association between prior health and survival in retirement over longer periods of time. Years spent in retirement is a way of describing this phenomenon and it can be calculated for cohorts with mortality follow-up until very old age.

Our aim was to increase the understanding of midlife CVD risk factors and general health as predictors for pre-retirement workforce participation and post-retirement survival; the former measured through age at retirement and calculation of work-loss years and the latter represented as survival in retirement. The rationale for including work-loss years was that it is uncertain whether previous findings are influenced by mortality before retirement. To study this, we assessed CVD risk factors and aspects of general health among Finnish male executives in midlife (mean age 47.8 years) and obtained information on their retirement status in the year 2000 and by that time the youngest participants had reached the age of 66 years. Thereafter, assessment of register-based all-cause mortality continued until the 9th decade in the year 2018. We hypothesized that greater CVD risk and poorer general health in midlife would associate with premature retirement and with poorer survival in retirement.

## Methods

### Study population

**Figure 1:**
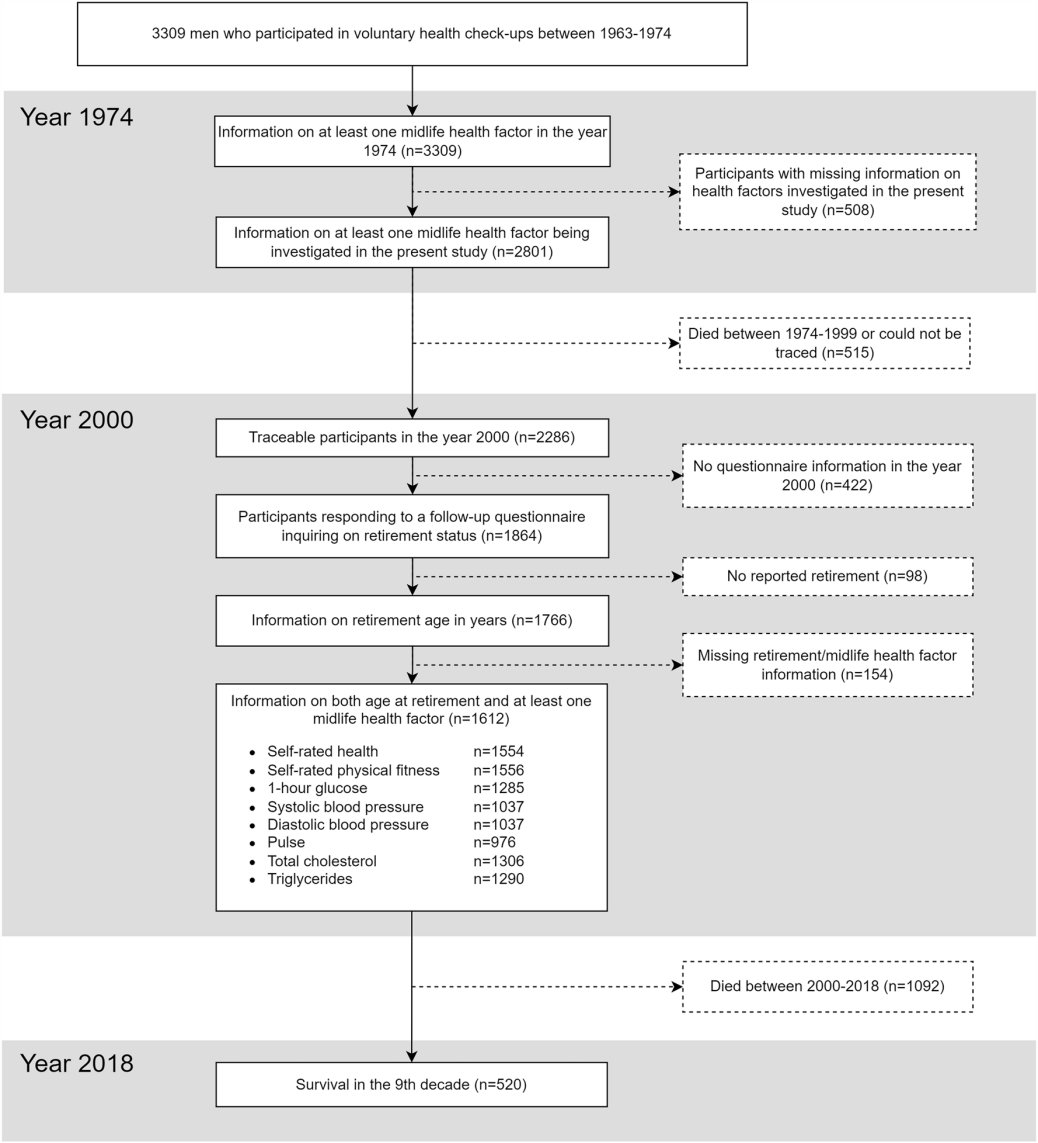
Flowchart over the 3,309 men and their inclusion in the present study. Solid lines represent groups relevant to the present study.

The Helsinki Businessmen Study is a longitudinal study of 3,490 initially healthy men who were born between 1919 and 1934^10^. The present sample (n = 3,309) consists of participants who could be traced using personal identification numbers assigned to all Finnish residents in the 1970s. Figure 1 presents a flowchart of the study population. The men (mean age 47.8 years in the year 1974) participated in voluntary health check-ups covering a range of cardiovascular health factors at the Finnish Institute of Occupational Health. This information was then used to calculate estimates of the participants’ 5-year risk of incident CVD^11^ and 10-year risk of incident fatal CVD^12^. Information on retirement was obtained when the youngest participants had reached the age of 66 years in the year 2000; out of 2,286 traceable participants, 1,864 returned the questionnaire. Full retirement data were available for 1,766 participants, of whom 1,612 also had information on midlife cardiovascular health factors. The participants’ deaths were tracked from The Digital and Population data services Agency’s Population Information System between the years 1974 and 2018.

### Cardiovascular health factors in 1974–1975

Midlife cardiovascular health factors, measured in the 1970s, included clinical and laboratory data^10^, such as blood pressure (BP), heart rate, fasting total cholesterol (TC)^13^, triglyceride levels (TG), and 1 h blood glucose levels after an oral glucose tolerance test (1 h-OGTT load)^14^. Participants' global self-rated health (SRH) and self-rated physical fitness (SRF) were assessed with the question, "What do you think about your present state of health/physical fitness; is it ‘very good’, ‘fairly good’, ‘average’, ‘fairly poor’ or ‘very poor’?" SRH categories 'fairly poor' and 'very poor' were combined into 'poor' due to the small number of men who perceived their SRH as 'very poor'.

We estimated the participants’ 5-year risk of incident CVD^11^ using a tool, that was developed among European and U.S. men aged 40–59 years free from coronary heart disease. The risk score^11^ uses information on age, systolic BP, serum TC, smoking status and body mass index (BMI). We also calculated the participants' 10-year composite risk score of fatal CVD in midlife and old age using the European 'low risk' SCORE-chart (Finnish)^12^. The chart gives estimates of the relative risk of fatal CVD events in a 10-year period. SCORE has been externally validated^15^ and uses sex, smoking status, systolic BP, and serum TC to estimate CVD risk.

### Covariates in the year 1974

In 1974, height and weight measurements were used to calculate BMI (kg/m2). Smoking status, based on self-reported data, was categorized as current smokers, ex-smokers, and never smokers. Similarly, self-reported weekly alcohol consumption in grams of pure alcohol was recorded.

### Retirement age and time spent in retirement evaluated in 2018

In 2000, participants reported their retirement status and age at retirement^16^. We excluded participants reporting no retirement (n = 98) and analysed data from 1766 men. Time spent in retirement was calculated in years from the onset of retirement until death or the end of follow-up in the year 2018. Approximately two thirds (n = 1,999, 67.9%) of the sample had died by 2018, and we observed 43,086 person-years of mortality follow-up from the onset of retirement.

### Working years lost due to death or early retirement

To study whether the inclusion of participants who died before retiring would affect our results, we calculated work-loss years^17^ as death or retirement occurring before the general age limit for old-age pensions. We used 65 years as the limit for old age pensions^17^, which in this cohort was also the most common age to retire (n = 250, 14.2%). The respective observations in person-years were 4,804 and 24,627, for deceased and those retiring younger than at age 65 years.

### Statistical methods

We present continuous variables as means and standard deviations and used one-way analyses of variance and Kruskall-Wallis tests for group central tendency statistics. Categorical data are presented as proportions. Covariate-adjusted Cox proportional hazard regression models and models extended for time-dependent effects were used to estimate hazard ratios (HRs) and 95% confidence intervals. For complex associations figures of HRs are shown. When the proportionality of hazards assumption is not met for covariate *Z*, the Cox- regression model can be extended to handle time-dependent effects by adding an effect of covariate interaction with a function of follow-up time. Thus, in our models the hazard function, *h*_*i*_(*t*), for participant *i* (*i* = 1, …, *N*) was specified as:

1 $$h_{i} (t) = h_{0} (t)e^{{\gamma 0z_{i} + \gamma tz_{i} g(t) + \beta^{{\text{T}}} {\text{x}}_{i} }} ,$$

where *h*_0_(*t*) is the baseline hazard function, *z*_*i*_ is the covariate value with a time-invariant and time-dependent effects *γ*0 and *γt*, respectively, *g*(*t*) is a suitable function of time, *t*, and $\beta$ is the vector of regression coefficients for other time-invariant fixed-effects for other covariates in vector **x**_*i*_. Notably, the effect of covariate *Z* is partitioned into two components: the time-invariant effect of the predictor and a time-varying quantifying dynamically the difference to the time-invariant effect over the follow-up. The function of time can take various functional forms, and we have indicated the selected function for each predictor in the tables for time-dependent effects. To get an idea of the total impact of covariate *Z*, the time-varying and time-invariant effects must be interpreted together, the HR estimates themselves are difficult to digest, and it is more informative to present the hazard ratio as a figure. In absence of time-dependent effects, the model Eq. (1) becomes the conventional proportional hazards Cox regression model.

As model validation, we assessed time-dependency in model effects using plots and tests based on scaled Schoenfeld-residuals, functional form of model effects based on martingale residuals, and impact from outliers based on dfbeta-statistics. Extended model effects (non-linear and time-dependent effects) are shown using predicted hazard plots. We adjusted our models with smoking, alcohol consumption, and continuous BMI in midlife. As some of these variables were used in estimating CVD risk, analyses of CVD risk were adjusted for alcohol consumption only. Supplementary Tables 1–2 show information about support for model assumptions and count of participants at risk in our analysis regarding age at retirement. Supplementary Tables 3–4 show the respective information in our analysis on survival in retirement. We normalized work-loss data by performing a log (base 10) transformation and back-transformed the point estimates. For the models we report hazard ratios or regression coefficients together with their 95% confidence intervals. The analyses were carried out using R software^18^ (version 4.2.1) with packages survival^19^ and extrafont^20^, and linear regression models in Stata (Release 17. College Station, TX: StataCorp LLC).

### Ethical approval

The research protocol of the follow-up study of HBS has been approved by the Ethics Committee of the Department of Medicine, University of Helsinki. The study adheres with the principles stated in the Declaration of Helsinki.

### Consent to participate

Informed consent was obtained from all individual participants included in the study.

## Results

### Pre-retirement workforce participation: age at retirement and work-loss years

The men had retired at the mean age of 61.2 years (SD = 4.4 years). Table 1 shows that men in the lowest quintile of retirement age (< 58 years) had had poorer SRH and SRF in midlife and lower mean risk of 5- and 10-year estimated risk of non-fatal/fatal CVD than those in the highest quintile retiring at age ≥ 65 years. In addition, 441 men had died before the statutory retirement age of 65 years. The combined work-loss through retiring or dying before age 65 years was 4.8 years (SD = 4.3 years; range = 0–38 years).

**Table 1:** Characteristics of the study population according to quintiles of age at retirement.

	Quintiles of age at retirement						
	N	0–20th percentile, ≤ 58y	20–40th percentile, 59-60y	40–60th percentile, 61-62y	60–80th percentile, 63-64y	80–100th percentile, ≥ 65y	*p*
Midlife characteristics in the year 1974							
Age, average years (SD)	1766	45.8 (3.7)	46.5 (3.9)	47.4 (3.9)	47.9 (3.8)	48.4 (4.2)	< 0.001
Self-rated health, %	1554						< 0.001
Very good	101	3.7	6.7	6.2	6.9	8.7	
Fairly good	711	38.7	46.0	44.5	50.2	48.9	
Average	667	49.7	39.6	46.6	40.6	39.0	
Poor	75	8.0	7.6	2.8	2.3	3.3	
Self-rated physical fitness, %	1556						0.002
Very good	56	1.0	4.9	3.1	3.3	5.4	
Good	490	32.6	29.3	25.3	34.1	35.7	
Fair	795	47.8	51.5	59.2	51.5	46.2	
Poor	200	16.3	13.7	12.1	10.8	11.4	
Very poor	15	2.3	0.6	0.3	0.3	1.2	
1 h-OGTT, mmol/l (SD)	1285	7.1 (2.1)	6.7 (1.8)	7.0 (1.9)	6.8 (1.9)	7.0 (2.1)	0.262
Systolic BP, mmHg (SD)	1037	142.7 (18.5)	140.7 (17.3)	142.2 (16.8)	141.1 (18.6)	142.7 (18.6)	0.775
Diastolic BP, mmHg (SD)	1037	93.1 (12.0)	90.3 (10.9)	91.6 (10.5)	90.2 (11.4)	91.3 (11.7)	0.179
Pulse, bpm (SD)	976	65.5 (10.7)	64.3 (10.0)	64.1 (11.1)	63.9 (10.7)	63.8 (10.3)	0.559
Total cholesterol, mmol/l (SD)	1306	6.3 (1.1)	6.1 (1.0)	6.2 (1.0)	6.1 (1.0)	6.2 (1.0)	0.464
Triglycerides, mmol/l (SD)	1290	1.7 (1.0)	1.6 (0.8)	1.6 (0.8)	1.5 (0.7)	1.5 (0.9)	0.028
Body mass index, kg/m^2^ (SD)	1357	26.1 (2.7)	26.1 (2.8)	25.8 (2.5)	25.3 (2.7)	25.6 (2.7)	0.002
Alcohol consumption, g/week (SD)	1371	168 (153)	163 (136)	140 (122)	136 (129)	145 (127)	0.026
Smoking status, %	1752						0.905
Current smoker		23.1	23.2	22.5	20.8	21.2	
Midlife 5-year risk of incident CVD, per SD	1006	0.38 (0.98)	0.37 (0.96)	0.48 (0.93)	0.42 (0.94)	0.63 (0.95)	0.018
Midlife SCORE 10-year risk of fatal CVD, % (SD)	1034	0.80 (0.93)	0.85 (0.98)	1.08 (1.21)	1.11 (1.40)	1.35 (1.56)	0.002
Categorical midlife SCORE relative risk of 10-year fatal CVD, n (%)	1034						0.003
< One fold	42	71 (42.0)	97 (42.9)	74 (37.2)	83 (38.8)	69 (30.5)	
1	543	77 (45.6)	86 (38.1)	75 (37.7)	75 (35.0)	94 (41.6)	
Two fold	201	11 (6.5)	28 (12.4)	27 (13.6)	32 (15.0)	26 (11.5)	
Three Four fold	145	8 (4.7)	14 (6.2)	18 (9.0)	17 (7.9)	23 (10.2)	
≥ Five fold	103	2 (1.2)	1 (0.4)	5 (2.5)	7 (3.3)	14 (6.2)	
Old-age characteristics in the year 2000							
Age, average years (SD)	1766	71.8 (3.7)	72.5 (3.9)	73.4 (3.9)	73.9 (3.8)	74.4 (4.2)	< 0.001
CVD, n (%)	1767	70.8	63.0	60.2	60.0	59.4	0.010
Old age SCORE 10-year risk of fatal CVD, % (SD)	1212	5.8 (2.6)	5.8 (2.4)	5.6 (2.0)	5.9 (2.2)	5.7 (2.5)	0.259
Survival characteristics							
Survival in retirement, years (SD)	1767	29.2 (6.9)	25.0 (6.1)	24.4 (5.5)	23.3 (5.6)	20.4 (5.7)	< 0.001
Mortality between 2000 and 2010, n (%)	1766	122 (35.7)	144 (38.6)	107 (33.0)	115 (33.3)	137 (35.9)	0.530
Mortality between 2011 and 2018, n (%)	1766	112 (32.7)	105 (28.2)	109 (33.6)	109 (31.6)	141 (36.9)	0.141

SD = standard deviation, BP = blood pressure, CVD = cardiovascular disease; OGTT = oral glucose tolerance test.

### Post-retirement survival

After the men had retired, their mean survival time was 24.4 years (SD = 6.6 years; range 3–54 years). The lexis diagram indicated that survival in retirement was relatively evenly distributed (Supplementary Fig. 1). Mortality increased during the study period according to the degree of estimated risk of CVD in midlife; in the year 2018 fewer than 1 out of 10 had survived in the groups with the highest risk, whereas slightly less than half in the low-risk groups were alive in the year 2018 (Supplementary Table 5).

### Health in midlife and sample selection

We report findings on men who had been measured for CVD health factors in midlife and who provided information on retirement age in a questionnaire (Fig. 1). Participants who had been assessed for CVD health factors in midlife but who were not included in the present study because of non-respondence to the questionnaire (n = 557) or death (n = 889) had been less healthy in most considered health factors in midlife (Supplementary Table 6).

### Self-rated health and physical fitness in midlife as determinants of pre-retirement workforce participation

Poorer SRH (average or poor vs. very good) and SRF (very good vs. fair) were associated with higher hazards of retiring at a younger age, of which poorer SRH (poor vs. very good, covariate-adjusted HR = 1.63, 95% CI = 1.14, 2.33) and SRF (very good vs. fair, covariate-adjusted HR = 0.71, 95% CI = 0.52, 0.94) remained statistically significant after adjusting for smoking, alcohol consumption, and BMI in midlife (Table 2). Similarly, men with poor SRH (poor vs. very good) had 0.32 years more work-loss (95% CI = 0.13, 0.52) in analyses adjusted with smoking, BMI, and alcohol consumption in midlife (Table 3). Poor SRF (very poor vs. fair) was initially associated with more work-loss (crude β = 0.45, 95% CI = 0.14, 0.66), but this association attenuated following adjustment for covariates (covariate-adjusted β = 0.38, 95% CI = − 0.29, 0.69).

**Table 2:** Cox regression models of midlife health factors as predictors of retirement age in years among older retired businessmen.

Predictor^a^	Crude		Adjusted^b^	
	Events	Crude HR (95% CI)	Events	Adjusted HR (95% CI)
Self-rated health	1533		1270	
Very good	101	Ref	90	Ref
Fairly good	702	1.20 (0.97, 1.48)	620	1.15 (0.92, 1.44)
Average	660	**1.32 (1.07, 1.62)**	513	1.19 (0.95, 1.49)
Poor	70	**1.80 (1.32, 2.44)**	47	**1.63 (1.14, 2.33)**
Self-rated physical fitness	1535		1272	
Very good	56	**0.69 (0.52, 0.90)**	53	**0.71 (0.52, 0.94)**
Good	479	0.93 (0.83, 1.05)	407	0.93 (0.82, 1.05)
Fair	789	Ref	659	Ref
Poor	197	1.05 (0.90, 1.23)	146	1.03 (0.86, 1.24)
Very poor	14	1.14 (0.67, 1.94)	7	0.79 (0.38, 1.67)
1 h-OGTT, mmol/l	1270	1.00 (0.98, 1.03)	1210	1.00 (0.97, 1.03)
Systolic BP, mmHg	1024		991	
- linear		**1.04 (1.00, 1.08)**		1.04 (1.00, 1.08)
- quadratic		**1.00 (1.00, 1.00)**		1.00 (1.00, 1.00)
Diastolic BP, mmHg	1024	1.00 (1.00, 1.00)	991	1.00 (1.00, 1.01)
Pulse, bpm	963	1.00 (1.00, 1.01)	899	1.00 (1.00, 1.01)
Total cholesterol, mmol/l	1291	1.02 (0.96, 1.07)	1231	1.02 (0.96, 1.07)
Triglycerides, mmol/l^c^	1275		1217	
- time-constant		0.99 (0.92, 1.07)		0.97 (0.89, 1.05)
- time-varying		**1.00 (1.00, 1.00)**		**1.00 (1.00, 1.00)**
Midlife 5-year risk of incident CVD, per SD	993	**0.91 (0.85, 0.97)**	987	**0.90 (0.84, 0.96)**
Midlife SCORE 10-year relative risk of fatal CVD	1021		988	
< 1	389	Ref	370	Ref
1	404	0.91 (0.79, 1.05)	393	0.90 (0.78, 1.03)
2	121	**0.79 (0.64, 0.97)**	120	**0.79 (0.64, 0.97)**
3–4	60	0.85 (0.65, 1.12)	59	0.85 (0.65, 1.11)
≥ 5	47	**0.54 (0.40, 0.73)**	46	**0.51 (0.30, 0.70)**

HR = hazard ratio, CI = confidence interval, BP = blood pressure, CVD = cardiovascular disease; OGTT = oral glucose tolerance test.

^a^Analyzed individually.

^b^Model adjusted with alcohol consumption, smoking, and body mass index in 1974. Models of midlife 5-year and 10-year risk of CVD were adjusted with alcohol consumption only.

^c^Mean-centered.

Significant values are in bold.

**Table 3:** Linear regression model estimates of midlife health factors as predictors of work-loss years among older business executives (back-transformed from log10).

Midlife health factors^a^	N	Crude	N	Adjusted^b^
		β (95% CI)		β (95% CI)
Self-rated health	1520		1256	
Very good		Ref		Ref
Fairly good		0.05 (-0.12, 0.19)		0.05 (-0.13, 0.21)
Average		0.13 (-0.03, 0.26)		0.11 (-0.06, 0.27)
Poor		**0.32 (0.16, 0.46)**		**0.32 (0.13, 0.52)**
Self-rated physical fitness	1522		1258	
Very good		-0.01 (-0.23, 0.19)		0.04 (-0.19, 0.25)
Good		0.02 (-0.06, 0.11)		0.06 (-0.04, 0.14)
Fair				
Poor		0.10 (-0.00, 0.21)		0.08 (-0.04, 0.20)
Very poor		**0.45 (0.14, 0.66)**		0.38 (-0.29, 0.96)
1 h-OGTT, mmol/l	1273	**0.02 (0.01, 0.04)**	1200	**0.01 (0.00, 0.03)**
Systolic BP, mmHg	965	**0.00 (0.00, 0.00)**	935	0.00 (-0.00, 0.00)
Diastolic BP, mmHg	965	**0.00 (0.00, 0.01)**	935	0.00 (-0.00, 0.01)
Pulse, bpm	890	0.00 (-0.00, 0.01)	830	0.00 (-0.00, 0.01)
Total cholesterol, mmol/l	1301	0.03 (-0.00, 0.07)	1222	0.03 (-0.00, 0.07)
Triglycerides, mmol/l	1284	**0.07 (0.03, 0.12)**	1208	**0.05 (0.01, 0.09)**
5-year risk of incident CVD, per SD	940	**-**0.00 (-0.05, 0.04)	933	-0.01 (-0.06, 0.04)
Midlife SCORE 10-year relative risk of fatal CVD	963			
< 1		Ref		Ref
1		**0.33 (0.15, 0.55)**		**0.32 (0.13, 0.53)**
2		**0.33 (0.15, 0.57)**		**0.31 (0.11, 0.54)**
3–4		0.19 (-0.06, 0.40)		0.16 (-0.09, 0.38)
≥ 5		0.17 (-0.09, 0.40)		0.16 (-0.11, 0.38)

Note. CI = confidence interval; BP = blood pressure; CVD = cardiovascular disease; OGTT = oral glucose tolerance test β = back-transformed point estimate.

^a^Analyzed individually.

^b^Adjusted for alcohol consumption, smoking, and body mass index in 1974. Models of midlife 5-year and 10-year risk of CVD were adjusted with alcohol consumption only.

Significant values are in bold.

### Laboratory and clinical CVD risk factors in midlife as determinants of pre-retirement workforce participation

**Figure 2:**
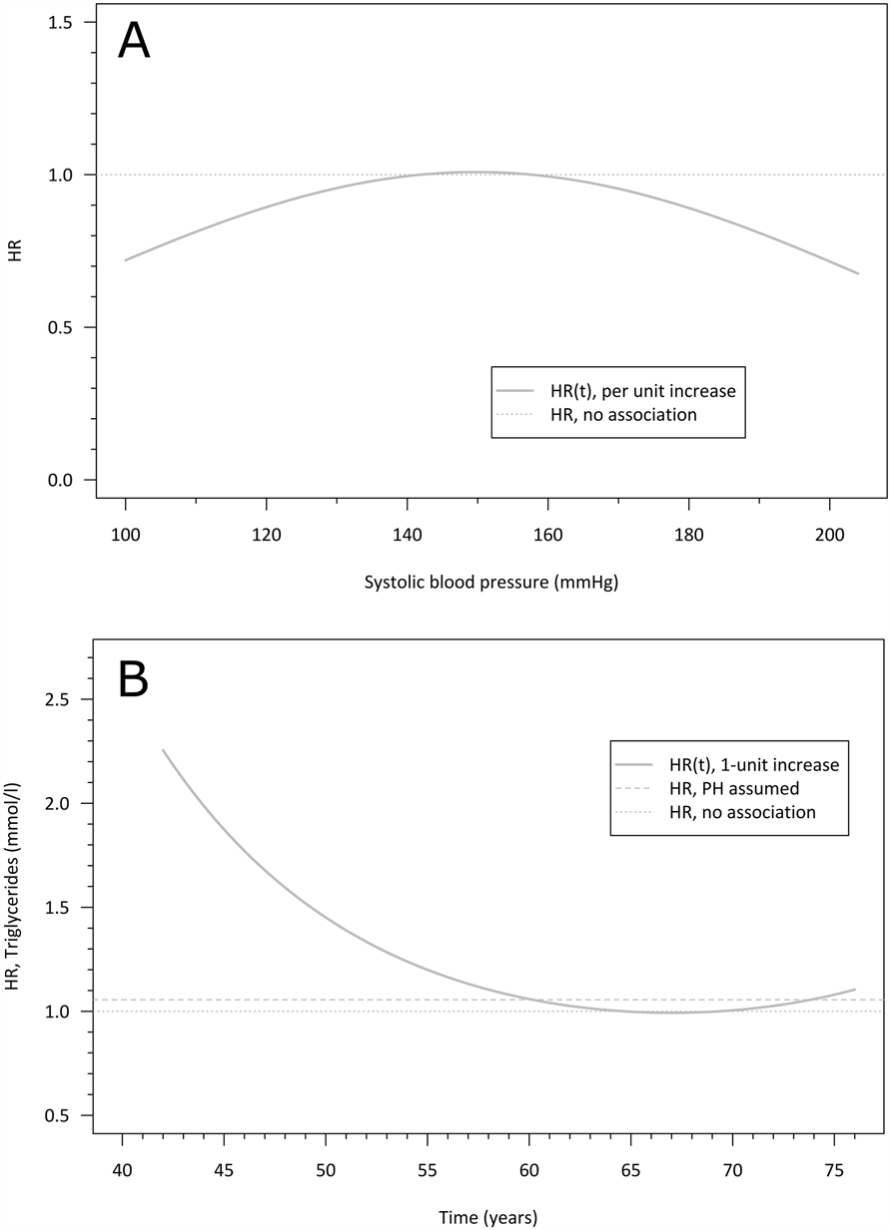
Predicted time-dependent hazard ratios for systolic blood pressure (panel **A**, top) and triglyceride concentration (panel **B**, bottom) assessed in midlife. The curves show the time-varying HR (solid dark grey line) of retirement age in years, and reference lines for HR estimated under the proportional hazards assumption (dashed grey line) and HR for strictly equal hazards (grey, dotted line).

Some but not all individual CVD risk factors showed associations with earlier retirement or more work-loss years, with many associations becoming statistically non-significant after covariate adjustment (Tables 2 and 3). Systolic BP had a curvilinear association to retirement hazard, suggesting that those in either low or high end of the range of observed readings had a lower hazard of retiring than those with readings in the mid-range (Fig. 2A). The association was weak, and it did not change substantially after covariate adjustment, which also reduced the sample size. Similarly, one-unit increases in systolic BP and diastolic BP showed initial positive associations of more work-loss years, but these associations attenuated after adjustment for smoking, BMI, and alcohol consumption (Table 3). Time-dependent hazards of unit-higher triglyceride levels showed an elevated hazard rate (HR(t) ~ 2.2) of earlier retirement, which then levelled off by age 60 years (Table 2 and Fig. 2B). For each 1 mmol/l increase in 1 h-OGTT (covariate-adjusted β = 0.01, 95% CI = 0.00, 0.03) and triglycerides (covariate-adjusted β = 0.05, 95% CI = 0.01, 0.09), the men were more likely to have more work-loss years (Table 3).

### Composite CVD risk estimates in midlife as determinants of pre-retirement workforce participation

The associations between midlife CVD risk and retirement and work-loss years were conflicting; while higher risk of CVD was associated with retiring at an older age, there was evidence suggesting that those with high risk were more likely to have more years of work-loss (Tables 2 and 3). Higher 5- and 10-year CVD risk scores in midlife were associated with a lower covariate-adjusted hazard of retirement (Table 2). Respectively for work-loss years, each increase of one standard deviation in the 5-year risk of incident CVD was not associated with work-loss years. The association between 10-year fatal CVD risk estimates and work loss showed that individuals with a 10-year fatal CVD risk estimate of 1 or higher vs. less than 1 relative risk had 0.32 more work-loss years (covariate-adjusted β = 0.32, 95% CI = 0.13, 0.53).

### Health in midlife as a determinant of post-retirement survival

**Figure 3:**
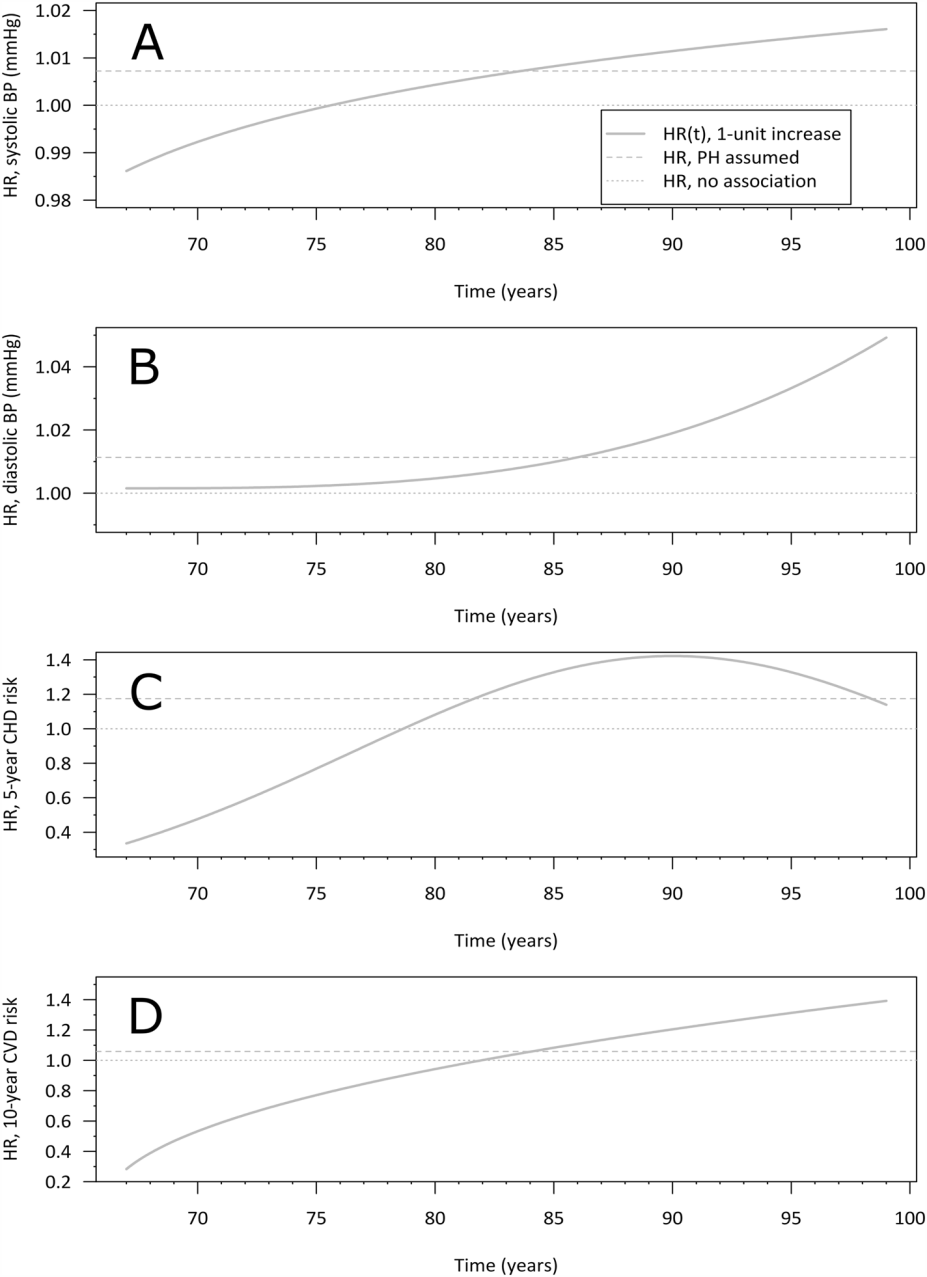
Predicted time-dependent hazard ratios for systolic blood pressure (panel **A**, top), diastolic blood pressure (panel **B**), 5-year risk of incident CVD (panel **C**), and 10-year risk of fatal CVD (panel **D**, bottom). The curves show the time-varying HR (solid dark grey line) of survival in years, and reference lines for HR estimated under the proportional hazards assumption (dashed grey line) and HR for strictly equal hazards (grey, dotted line).

Poorer SRH (average or poor vs. very good) and SRF (poor or very poor vs. fair) were associated with fewer years spent in retirement, of which poor SRH (poor vs. very good, covariate-adjusted HR = 2.05, 95% CI = 1.37, 3.07) and SRF (poor vs. fair, covariate-adjusted HR = 1.26, 95% CI = 1.20, 1.55) remained after adjustment for smoking, alcohol consumption, and BMI in midlife (Table 4). Per each 1 mmol/l higher total cholesterol (covariate-adjusted HR = 1.08, 95% CI = 1.01, 1.15) and triglycerides (covariate-adjusted HR = 1.08, 95% CI = 1.01, 1.15) the men were more likely to spend fewer years on retirement (Table 4). Higher systolic BP, diastolic BP, and midlife cardiovascular risk scores showed age-adjusted time-dependent hazard of spending years in retirement (Table 4 and Fig. 3). In panel A, unit-higher midlife systolic BP was associated with an almost linearly increasing hazard from age 75 years to HR(t) ~ 1.015 at age 97. In panel B, unit-higher midlife diastolic BP showed an exponential rate of growth in the time-dependent hazard rate of fewer years spent in retirement from age 67 years with a peak of HR(t) ~ 1.05 at age 97. In panel C, one standard deviation higher estimated 5-year incident risk of cardiovascular disease in midlife showed curvilinear time-dependent elevated hazard rates peaking at approximately age 85 years (HR(t) ~ 1.3)., which levelled off so that the hazard rate remained indicative of elevated hazard for higher risk scores. In panel D, unit-higher 10-year risk of cardiovascular disease showed a linear increase from age 82 years to HR(t) ~ 1.4 at age 97.

**Table 4:** Cox regression models of midlife health factors as predictors of survival in retirement among older retired businessmen.

Predictor^a^	N	Crude		N	Adjusted^f^	
		Events	HR (95% CI)		Events	HR (95% CI)
Self-rated health	1554	1053		1287	873	
Very good	101	58	Ref	90	55	Ref
Fairly good	711	465	1.31 (0.99, 1.72)	628	418	1.21 (0.91, 1.60)
Average	667	466	**1.42 (1.08, 1.87)**	519	357	1.16 (0.87, 1.54)
Poor	75	64	**2.46 (1.72, 3.51)**	50	43	**2.05 (1.37, 3.07)**
Self-rated physical fitness	1556	1053		1289	874	
Very good	56	34	0.79 (0.56, 1.12)	53	33	0.93 (0.65, 1.33)
Good	490	327	0.92 (0.81, 1.07)	417	284	1.02 (0.87, 1.18)
Fair	795	527	Ref	664	440	Ref
Poor	200	153	**1.39 (1.16, 1.67)**	148	112	**1.26 (1.20, 1.55)**
Very poor	15	12	**2.04 (1.15, 3.62)**	7	5	1.31 (0.54, 3.19)
1-h glucose, mmol/l	1285	875	**1.04 (1.01, 1.08)**	1335	842	1.02 (0.99, 1.06)
Systolic BP, mmHg	1037	690		1004	673	
- time-constant			**0.95 (0.91, 1.00)**			**0.95 (0.90, 1.00)**
- time-dependent			**1.02 (1.00, 1.03)**			**1.02 (1.00, 1.03)**
Diastolic BP, mmHg	1037	690		1004	673	
- time-constant			1.00 (0.99, 1.01)			0.94 (0.87, 1.01)
- time-dependent			**1.02 (1.00, 1.04)**^**d**^			**1.02 (1.00, 1.05)**^**d**^
Pulse, bpm	976	656	1.00 (1.00, 1.01)	910	614	1.00 (0.99, 1.01)
Total cholesterol, mmol/l	1306	887	**1.14 (1.07, 1.21)**	1246	855	**1.08 (1.01, 1.15)**
Triglycerides, mmol/l	1290	875	**1.13 (1.06, 1.22)**	1246	855	**1.08 (1.01, 1.15)**
Midlife 5-year risk of incident CHD, per SD^b^	1006	673		1000	671	
- time-constant			**1.42 (1.28, 1.58)**			**1.40 (1.26, 1.55)**
- time-dependent			**0.76 (0.69, 0.84)**^**e**^			**0.76 (0.68, 0.83)**^**e**^
Midlife SCORE 10-year risk of fatal CVD	1034	689		1001	672	
- time-constant			**0.28 (0.17, 0.47)**			**0.28 (0.17, 0.47)**
- time-dependent			**1.58 (1.33, 1.87)**^**d**^			**1.58 (1.33, 1.87)**
Old age SCORE risk of fatal CVD	1212	810	**1.05 (1.02, 1.08)**	929	624	**1.04 (1.00, 1.07)**

HR = hazard ratio, CI = confidence interval, BP = blood pressure, CVD = cardiovascular disease.

^a^Analyzed individually. ^b^Log-transformed. ^c^Log-transformed, centered time. ^d^Cube-transformed, rescaled time. ^e^Squared, rescaled time. ^f^Model adjusted with alcohol consumption, smoking, and body mass index in 1974. Models of midlife 5-year and 10-year risk of CVD were adjusted with alcohol consumption only.

Significant values are in bold.

## Discussion

Cardiovascular disease (CVD) is a leading cause of disability pension and premature exit from the workforce, significantly affecting work ability and labour force participation^2,5^. Despite its impact, limited research has focused on studying CVD risk factors or composite CVD risk estimates in populations without pre-existing CVD. Our study, based on business executives followed for up to 44 years, found a time-dependent relationship between higher triglyceride levels in midlife and earlier retirement, independent of lifestyle-related factors including smoking, alcohol use, and BMI in midlife. Furthermore, although men with higher CVD risk in midlife were more likely to retire at an older age, we found evidence to support that they also experienced more work-loss years, i.e., to retire or die before age 65 years. After the participants had retired, those who had had higher CVD risk in their midlife also had higher mortality risk in retirement. Poorer self-rated health and physical fitness predicted earlier retirement, more work-loss years, and poorer survival in retirement.

### Interpretation in light of known literature

We were able to expand the previous scarce literature on CVD risk and retirement, where both early and on-time retirees displayed similar CVD risk factors^7^, by providing evidence of higher triglyceride levels associating with increased hazard of earlier retirement. While this association was independent of lifestyle factors including alcohol consumption, higher triglyceride levels could still reflect overall poor health, stress, or other factors that predispose to early retirement. Poorer self-rated health, a known predictor of transitioning into disability pensions^21–23^, was also associated with increased hazard of earlier retirement in our study, thus corroborating previous findings. Individuals who self-rate their health as favourable may exhibit improved work ability and the absence of conditions which may compromise their ability to work^21–23^. However, our results suggest that CVD risk may not be a major determinant of earlier retirement overall, as most other CVD risk factors were unrelated to hazard of earlier retirement. CVD risk alone may not be sufficient to generate symptoms with significant impact work ability. Moreover, men at higher risk of developing CVD in midlife were not at increased hazard of retiring early; in fact, they showed a decreased hazard. Considering that the men underwent clinical examinations in the Finnish Institute of Occupational Health and that by old age the difference in CVD risk by retirement age appeared to have levelled off, it is possible that men with high CVD risk received interventions to mitigate their high risk.

Considering these findings and the fact that one can exit the workforce also through premature mortality, we found parallel evidence to support CVD risk factors as determinants of more work-loss years, where participants retiring or dying prematurely (younger than age 65 years) were considered. We could expand the previous literature on estimated productivity losses due to medical conditions, e.g. hypertension^24,25^, heterozygous familial hypercholesterolemia^26^, and diabetes^27,28^ to include CVD risk factors and estimated CVD risk by showing evidence that higher CVD risk, levels of 1 h-OGTT, and serum triglycerides in midlife were associated with more work-loss years. However, as with retirement age, most other CVD risk factors were unrelated to work-loss, and findings on CVD risk and work-loss years were less consistent.

Our follow-up extended to the 9th decade, enabling us to describe survival among the study participants and provide valuable information on the impact of CVD risk factors in old age. While CVD risk factors are known predictors of overall survival^13,29,30^, a meta-analysis on the association between retirement age and survival^9^ highlighted the significance of prior health in their analysis. Our findings contribute to this gap by suggesting that midlife CVD health factors not only potentially impact productivity losses but also contribute to fewer years in retirement. We found that men with higher CVD risk factors, higher estimated risk of 5-year incident or 10-year fatal CVD, and poorer self-rated health and fitness, had a higher hazard for all-cause mortality in retirement, regardless of smoking, alcohol consumption, and BMI in midlife. Some of these associations also showed time-dependent hazards^9,13,17,24–30^.

### Importance of the results

These findings highlight the impact of CVD risk on the work-life trajectory of individuals and emphasize the importance of early identification and management of modifiable risk factors to reduce CVD-related morbidity and mortality. First, although CVD is a leading cause of premature retirement, we found no evidence to support CVD risk factors as major determinants of earlier retirement, except for triglyceride levels, self-rated health, and physical fitness. However, when premature mortality before age 65 years was also considered in combination with earlier retirement in work-loss years, greater overall CVD risk also emerged as a potential factor in productivity losses. Second, our results suggest that potential productivity losses related to higher CVD risk may also occur in healthy individuals, as only a small proportion of our study participants had CVD or diabetes in midlife. Therefore, efficient management of CVD risk may prevent potential productivity losses even among healthy individuals. Third, our study supports the notion that both general health and CVD risk factors are critical factors to consider when examining retirement outcomes and survival in general.

### Strengths and limitations

We examined the association between CVD risk factors, retirement age, work-loss years, and mortality in older male executives. The study followed the men for up to 44 years, until the 9th decade of life, and used national register-linkage to gather data on mortality. The sample was subject to selective survival and loss to follow-up, leaving out participants who had poorer general and CVD health in midlife, thus potentially undermining our results regarding retirement age and survival in retirement. Given that CVD is one of the leading causes of premature exit from the workforce^2,5^, we believe that participants at high risk of CVD but who did not survive to retirement age may have also been more likely to retire early due to CVD-related health problems. The study population consisted of Caucasian men from higher socioeconomic groups^10^, which provides internal validity but significantly limits generalizability to other socioeconomic groups, women, or individuals with different retirement policies. The men worked as executives and entrepreneurs who did not have fixed retirement ages. Laboratory CVD risk factors were measured in the 1970s using methods from that time, which may be outdated, but have been shown to correlate with clinical outcomes in later studies from this cohort^14^. Due to the extended time span between the assessment of CVD risk factors and retirement, participants' cardiovascular risk levels may have undergone changes or interventions, such as pharmacological treatments. Our definition of work-loss years has potential caveats as the retirement characteristics were self-reported. Nonetheless, we used a cut-off at 65 years, which has been used by others^17^.

### Future recommendations

Efforts aimed at early identification and management of CVD risk factors may not only enhance the productivity of individuals during their working years but also contribute to a longer and healthier retirement period. To achieve this, clinicians should prioritize assessing and addressing CVD risk factors in midlife to reduce morbidity and mortality. Targeted interventions should be implemented for individuals with poorer self-rated health, while comprehensive retirement planning should incorporate cardiovascular health considerations to promote a longer and healthier retirement period. Further investigation is warranted to explore the underlying mechanisms behind the association between higher triglyceride levels and the time-dependent hazard of early retirement, as the precise mechanisms remain unclear.

### Conclusions

This study found evidence that CVD risk factors can serve as predictors of both pre-retirement workforce participation and post-retirement survival. Although high midlife CVD risk was not a consistent predictor of earlier retirement, high risk may contribute to premature mortality, potentially leading to poorer pre-retirement workforce participation. Of considered CVD risk factors, only high midlife triglyceride levels were associated with the hazard to retire earlier. Midlife CVD health factors and composite risk estimates were linked to a higher hazard of mortality in retirement. Those with poorer self-rated health or fitness were at higher hazard to retire earlier, more likely to have more work-loss years, and at higher hazard for mortality in retirement, rendering them potential targets for interventions. The effective management of CVD risk factors may enhance the productivity of working-aged individuals and the survival of retirees.

### Supplementary Information

Supplementary Tables.

Supplementary Figure 1.

## Data Availability

The datasets used and/or analysed during the current study available from Timo E. Strandberg on reasonable request.
