# Supplementary material for: Midlife cardiovascular health factors as predictors of retirement age, work-loss years, and years spent in retirement among older businessmen
Source: Sci Rep. 2023 Oct 2;13:16526. doi: 10.1038/s41598-023-43666-x (PMC10545670; doi:10.1038/s41598-023-43666-x)
Supplement: Supplementary file 1 — Supplementary Tables. [file 41598_2023_43666_MOESM1_ESM.docx]

Supplementary Material

Midlife cardiovascular health factors as predictors of retirement age, work-loss years, and years spent in retirement among older businessmen

**Supplementary Table 1**. Results for assessment of crude Cox regression model assumptions in analyses of time to retirement age.

**Supplementary Table 2**. Count of participants at risk at start of period, and cumulative number of deaths and censorings at end of interval within grouped time intervals in the study.

**Supplementary Table 3**. Results for assessment of crude Cox regression model assumptions in analyses of time in retirement.

**Supplementary Table 4.** Count of participants at risk at start of period, and cumulative number of deaths and censorings at end of interval within grouped time intervals in the study.

**Supplementary Table 5.** Cumulative mortality according to groups of 5-year incident and 10-year fatal CVD risk scores assessed in midlife.

**Supplementary Table 6.** Midlife health factors of study participants responding vs. not responding to a questionnaire sent in the year 2000.

**Supplementary Figure 1**. Lexis diagrams of years spent in retirement according to A) retirement age in years and B) according to age in the year 2000.

**Supplementary Table 1**. Results for assessment of crude Cox regression model assumptions in analyses of time to retirement age.

| **Predictor** | **Global test of PH-violation,**  **p-value** | **Functional form,**  **p-value (LRT)** | **Influential observations (IO)** | |
| --- | --- | --- | --- | --- |
|  |  |  | **N** | **Impact of not excluding**  **IO on strength of HR** |
| SRH | **0.027**^a^ | N/A | 5 | Increase (all) |
| SRF | 0.149^b^ | N/A | **7** | Increase all,  but decrease very good/good |
| Glucose | 0.481 | Qua/**Lin**, 0.478 | 3 | Decrease |
| Systolic BP | 0.679 | **Qua**/Lin, 0.043 | **4** | Increase |
| - Qua model | 0.216 | **--** | **4** | Increase |
| Diastolic BP | 0.728 | Qua/**Lin**, 0.497 | 1 | Increase |
| Pulse | 0.242 | Qua/**Lin**, 0.839 | **4** | Decrease |
| Cholesterol | 0.394 | Qua/**Lin**, 0.411 | 2 | Decrease |
| Triglyc. | **0.036** | Qua/**Lin**, 0.077 | 5 | Decrease |
| 5-year CVD risk | 0.485 | **Lin** | 1 | Decrease |
| 10-year CVD risk | 0.112 | N/A | **13** | All increase, all HRs  n.s. if IO deleted |
| CVD risk, old age | N/A | N/A | N/A | N/A |

Note. LRT = likelihood-ratio test; SRH = self-rated health; SRF = self-rated fitness; BP = blood pressure; CVD = cardiovascular disease. Lin, linear effect (x), Qua, Lin + quadratic effect (x + x^2^). Results for the diagnostics for the adjusted models were similar and, hence, are not shown.

^a^Time-dependent effects were not substantial and hence not modelled.

^b^For the comparison ‘very good’ vs. ‘good’, p = 0.029; residual analysis using estimated dfbeta-values suggests the effect would be no longer statistically significant (p = 0.109) if outliers were removed.

**Supplementary Table 2**. Count of participants at risk at start of period, and cumulative number of deaths and censorings at end of interval within grouped time intervals in the study.

| **Period (years)** | |  | **Within time-interval** | |
| --- | --- | --- | --- | --- |
| **start** | **end** | **At risk** | **Events** | **Censorings** |
| [42, | 45] | 147 | 1 | 0 |
| (45, | 50] | 147 | 1 | 0 |
| (50, | 55] | 1192 | 20 | 0 |
| (55, | 60] | 1623 | 98 | 0 |
| (60, | 65] | 1326 | 572 | 0 |
| (65, | 70] | 382 | 919 | 0 |
| (70, | 75] | 35 | 111 | 0 |
| (75, | 76] | 2 | 21 | 0 |
| Total | | | 1742 | 0 |

**Supplementary Table 3**. Results for assessment of crude Cox regression model assumptions in analyses of time in retirement.

| **Predictor** | **PH-violation,**  **p-value** | **Functional form,**  **p-value (LRT)** | **Influential observations**  **N** |
| --- | --- | --- | --- |
| SRH | 0.863 | N/A | 0 |
| SRF | 0.395 | N/A | 3 |
| Glucose | 0.570 | Qua/**Lin**, 0.337 | 2 |
| Systolic BP | **0.020^b^** | **Lin** | 1 |
| Diastolic BP | **0.021^c^** | **Lin** | 3 |
| Pulse | 0.130 | Cub/**Lin**, 0.506 | 1 |
| Cholesterol | 0.701 | Cub/**Lin**, 0.189 | 3 |
| Triglyc. | 0.187 | Cub/**Lin**, 0.089 | 2 |
| 5-year CVD risk^a^ | **<0.001^d^** | **Lin** | 5 |
| 10-year CVD risk | **<0.001^b^** | Cub/**Lin**, 0.567 | 4 |
| CVD risk, old age | 0.291 | Cub/**Lin**, 0.068 | 2 |

Note. LRT = likelihood-ratio test. ^a^Log-transformed. ^b^Log-transformed centered time. ^c^Cube-transformed rescaled time. ^d^Square-transformed rescaled time. Bold typeface indicates statistically significant p-value at 0.05 level of significance, and functional form selection. Influential observations led to minor changes in the models, mainly increasing the hazard ratios. Functional form was tested based on the linear, quadratic, and cubic polynomial effect. Functional form test is reported for the comparison with the lowest p-value.

**Supplementary Table 4.** Count of participants at risk at start of period, and cumulative number of deaths and censorings at end of interval within grouped time intervals in the study.

| **Period (years)** | |  | **Within time-interval** | |
| --- | --- | --- | --- | --- |
| **start** | **end** | **At risk** | **Events** | **Censorings** |
| [ | 67] | 1682 | 1 | 0 |
| (67, | 70] | 1720 | 19 | 0 |
| (70, | 75] | 1681 | 90 | 0 |
| (75, | 80] | 1480 | 222 | 0 |
| (80, | 85] | 1122 | 371 | 77 |
| (85, | 90] | 425 | 349 | 304 |
| (90, | 95] | 73 | 134 | 153 |
| (95, | 98] | 10 | 15 | 31 |
| Total | | | 1201 | 565 |

**Supplementary Table 5.** Cumulative mortality according to groups of 5-year incident and 10-year fatal CVD risk scores assessed in midlife.

|  |  | **Categories of SCORE 10-year relative risk of fatal cardiovascular disease in the year 1974^a^** | | | | | | | | |  |
| --- | --- | --- | --- | --- | --- | --- | --- | --- | --- | --- | --- |
|  | Deaths | <1 | | 1 | | 2 | | 3-4 | | ≥5 | p |
|  |  |  | |  | |  | |  | |  |  |
| Died between 1974-1999, n (%) | 371 | 71 (12.9) | | 152 (23.4) | | 55 (26.3) | | 66 (37.5) | | 27 (43.5) | <0.001 |
| Cumulative mortality between 1974-2010, n (%) | 828 | 182 (33.2) | | 336 (51.7) | | 130 (62.2) | | 128 (72.7) | | 52 (83.9) | <0.001 |
| Cumulative mortality between 1974-2018, n (%) | 1223 | 314 (57.2) | | 502 (77.2) | | 185 (88.5) | | 162 (92.0) | | 60 (96.8) | <0.001 |
|  | | | | | | | | | | | |
|  |  | **Quintiles of Keys’ 5-year risk of incident cardiovascular disease in the year 1974** | | | | | | | | |  |
|  | Deaths | 0-20^th^ percentile | 20-40^th^ percentile | | 40-60^th^ percentile | | 60-80^th^ percentile | | 80-100^th^ percentile | | p |
|  |  |  |  | |  | |  | |  | |  |
| Died between 1974-1999, n (%) | 369 | 38 (11.8) | 62 (19.2) | | 64 (19.9) | | 79 (24.5) | | 126 (39.1) | | <0.001 |
| Cumulative mortality between 1974-2010, n (%) | 814 | 102 (31.7) | 136 (42.1) | | 163 (50.6) | | 178 (55.1) | | 235 (73.0) | | <0.001 |
| Cumulative mortality between 1974-2018, n (%) | 1203 | 175 (54.3) | 224 (69.3) | | 244 (75.8) | | 265 (82.0) | | 295 (91.6) | | <0.001 |

**Supplementary Table 6.** Midlife health factors of study participants responding vs. not responding to a questionnaire sent in the year 2000.

|  | **Individuals responding or not responding to the questionnaire sent in the year 2000** | |  |  |  |
| --- | --- | --- | --- | --- | --- |
|  | Provided occupational information in 2000 | Did not provide information on occupational status in 2000 |  | **Individuals who died during 1974-1999** |  |
|  | N=1864 | N=557 |  | N=889 |  |
| **Midlife health factors** | | |  |  |  |
| Self-rated health |  |  |  |  | <0.001 |
| Very good | 107 (6.5) | 24 (6.9) |  | 38 (5.4) |  |
| Fairly good | 752 (45.8) | 153 (43.6) |  | 261 (36.9) |  |
| Average | 703 (42.9) | 150 (42.7) |  | 332 (47.0) |  |
| Poor | 78 (4.8) | 24 (6.8) |  | 76 (10.7) |  |
| Self-rated physical fitness |  |  |  |  | <0.001 |
| Very good | 65 (4.0) | 9 (2.6) |  | 28 (4.0) |  |
| Good | 515 (31.4) | 109 (31.0) |  | 167 (23.6) |  |
| Fair | 838 (51.0) | 175 (49.9) |  | 360 (50.9) |  |
| Poor | 209 (12.7) | 57 (16.2) |  | 149 (21.1) |  |
| Very poor | 15 (0.9) | 1 (0.3) |  | 3 (0.4) |  |
| 1-hour glucose, mmol/l | 6.9 (2.0) | 7.1 (2.2) |  | 7.6 (2.5) | <0.001 |
| Systolic BP, mmHg | 141.6 (17.9) | 144.0 (20.9) |  | 146.6 (20.0) | <0.001 |
| Diastolic BP, mmHg | 91.1 (11.3) | 92.4 (11.3) |  | 93.9 (11.2) | <0.001 |
| Pulse, bpm | 64.2 (10.5) | 65.4 (11.3) |  | 66.9 (11.3) | <0.001 |
| Total cholesterol, mmol/l | 6.2 (1.0) | 6.3 (1.0) |  | 6.4 (1.1) | <0.001 |
| Triglycerides, mmol/l | 1.6 (0.8) | 1.7 (0.9) |  | 1.8 (1.1) | <0.001 |
| Midlife 5-year risk of incident CVD, per SD | 0.4 (1.0) | 0.6 (1.0) |  | 0.9 (1.0) | <0.001 |
| Midlife SCORE 10-year risk of fatal CVD | 2.1 (2.0) | 2.4 (2.4) |  | 3.2 (2.9) | <0.001 |

Note. BP=blood pressure; SD=standard deviation; CVD=cardiovascular disease.
