# Supplementary figures and images for: Midlife cardiovascular health factors as predictors of retirement age, work-loss years, and years spent in retirement among older businessmen
Source: Sci Rep. 2023 Oct 2;13:16526. doi: 10.1038/s41598-023-43666-x (PMC10545670; doi:10.1038/s41598-023-43666-x)

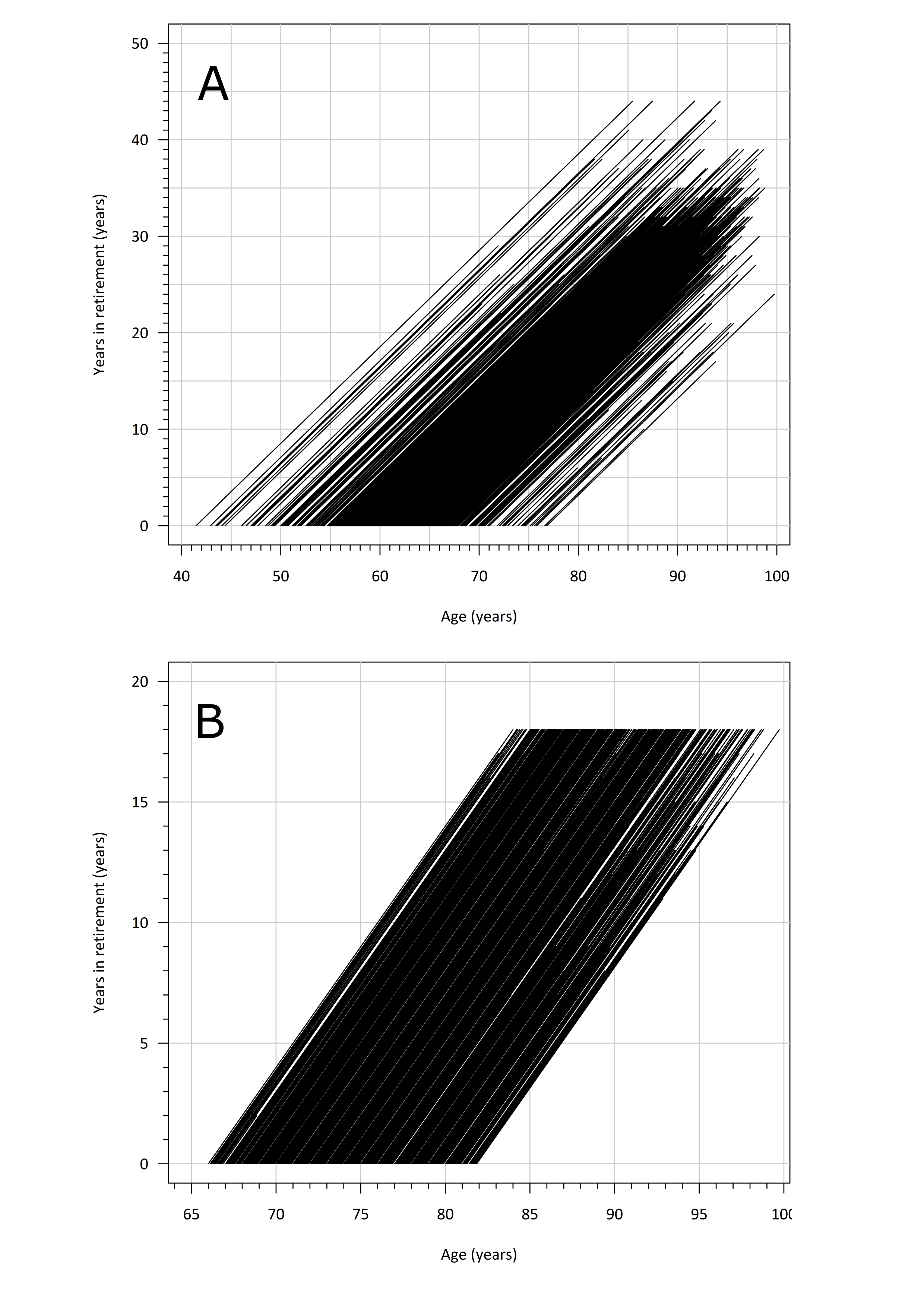

Supplement: Supplementary file 2 — Supplementary Figure 1. [file 41598_2023_43666_MOESM2_ESM.png]
